# Pathological complete response in multimodal treatment of esophageal cancer: a retrospective cohort study

**DOI:** 10.1093/dote/doac095

**Published:** 2022-12-27

**Authors:** Julian Hipp, Jasmina Kuvendjiska, Hans Christian Hillebrecht, Sylvia Timme-Bronsert, Stefan Fichtner-Feigl, Jens Hoeppner, Markus K Diener

**Affiliations:** Department of General and Visceral Surgery, Medical Center - University of Freiburg, Hugstetter Str. 55, 79106 Freiburg, Germany; Department of General and Visceral Surgery, Medical Center - University of Freiburg, Hugstetter Str. 55, 79106 Freiburg, Germany; Department of General and Visceral Surgery, Medical Center - University of Freiburg, Hugstetter Str. 55, 79106 Freiburg, Germany; Institute for Surgical Pathology, Medical Center - University of Freiburg, Faculty of Medicine - University of Freiburg, Breisacher Str. 115A, 79106 Freiburg, Germany; Department of General and Visceral Surgery, Medical Center - University of Freiburg, Hugstetter Str. 55, 79106 Freiburg, Germany; Department of Surgery, University Medical Center Schleswig-Holstein, UKSH Campus Lübeck, Ratzeburger Allee 160, 23538 Lübeck, Germany; Department of General and Visceral Surgery, Medical Center - University of Freiburg, Hugstetter Str. 55, 79106 Freiburg, Germany

**Keywords:** esophagogastric junction cancer, esophageal cancer, visceral surgery, oncologic surgery, pathologic complete response, multimodal treatment, neoadjuvant treatment, chemotherapy, chemoradiation

## Abstract

To evaluate pathological complete response (pCR, ypT0ypN0) after neoadjuvant treatment compared with non-complete response (non-CR) in patients with esophageal cancer (EC), and 393 patients were retrospectively analyzed. Survival probability was analyzed in patients with: (i) pCR vs non-CR; (ii) complete response of the primary tumor but persisting lymphatic metastases (non-CR-T0N+) and (iii) pCR and tumor-free lymphnodes exhibiting signs of postneoadjuvant regression vs. no signs of regression. (i) Median overall survival (mOS) was favorable in patients with pCR (pCR: mOS not reached vs. non-CR: 41 months, *P* < 0.001). Multivariate analysis revealed that grade of regression was not an independent predictor for prolonged survival. Instead, the achieved postneoadjuvant TNM-stage (T-stage: Hazard ratio [HR] ypT3-T4 vs. ypT0-T2: 1.837; N-stage: HR ypN1-N3 vs. ypN0: 2.046; Postneoadjuvant M-stage: HR ypM1 vs. ycM0: 2.709), the residual tumor (R)-classification (HR R1 vs. R0: 4.195) and the histologic subtype of EC (HR ESCC vs. EAC: 1.688) were prognostic factors. Patients with non-CR-T0N+ have a devastating prognosis, similar to those with local non-CR and lymphatic metastases (non-CR-T + N+) (non-CR-T0N+: 22.0 months, non-CR-T + N-: mOS not reached, non-CR-T + N+: 23.0 months; *P*-values: non-CR-T0N+ vs. non-CR-T + N-: 0.016; non-CR-T0N+ vs. non-CR-T + N+: 0.956; non-CR-T + N- vs. non-CR-T + N+: <0.001). Regressive changes in lymphnodes after neoadjuvant treatment did not influence survival-probability in patients with pCR (mOS not reached in each group; EAC-patients: *P* = 0.0919; ESCC-patients: *P* = 0.828). Particularly, the achieved postneoadjuvant ypTNM-stage influences the survival probability of patients with EC. Patients with non-CR-T0N+ have a dismal prognosis, and only true pathological complete response with ypT0ypN0 offers superior survival probabilities.

## INTRODUCTION

**Fig. 1:**
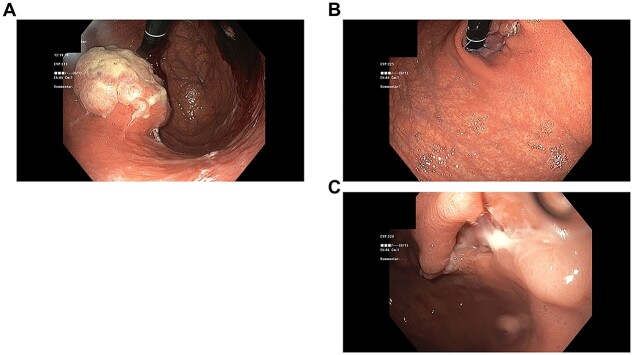
Endoscopic aspect of an AEG Type II-cancer. (A) Exophytic lesion prior to neoadjuvant treatment (Clinical Staging: uT3uN+). (B) and (C) Preoperative endoscopic view after four cycles of neoadjuvant chemotherapy according to FLOT-protocol. Only a residual ulcerous lesion can be seen at the esophagogastric junction. Postoperative histopathological result following 2-Field-Esophagectomy with gastric tube-reconstruction: ypT0ypN0.

Esophageal cancer (EC) shows a steadily increasing incidence in the western world and therefore exhibits a growing socio-economic impact.^1^ Worldwide in 2008, 3.955.919 disability-adjusted life years (DALYs) were attributed to EC. Years of life lost accounted for 97% of DALYs, while disability accounted for 3%.^2^ Standard treatment for locally advanced EC, which includes most EC-cases in western countries, is neoadjuvant chemotherapy (nCT) for Esophageal Adenocarcinoma (EAC) or neoadjuvant chemoradiation (nCRT) for EAC or Esophageal Squamous Cell Carcinoma (ESCC) followed by surgical resection either with transhiatal-extended gastrectomy or partial esophagectomy with gastric tube reconstruction.^3^^,^^4^ Neoadjuvant treatment has become increasingly effective and with modern nCT- or nCRT-protocols, pathological complete response (pCR) is detected in the surgical specimens in up to 16–35% in EAC-patients and up to 49% in ESCC-patients (Fig. 1).^5–7^

pCR is of prognostic significance in breast-, rectal-, and bladder cancer-patients. ^8–10^ Whether pCR after neoadjuvant treatment is an independent prognostic factor for EC-patients is under current discussion. A meta-analysis of seven studies with a total of 1143 treated patients—but only 77 patients with pCR—reported a significant improvement of overall survival in patients with pCR.^11^ This result has to be interpreted carefully, as it reflects the results of univariate analyses of the included studies. Individual study data are inconclusive as some studies report improved overall survival probability in patients with pCR,^12^^,^^13^ while others found that the independent predictor of survival probability was the achieved postneoadjuvant/postoperative ypTNM-stage ^14^ instead of the grade of pathological tumor response.^15^

Regarding the limited and inhomogeneous available clinical data, the aims of this study were to evaluate the clinical significance of pCR in our high-volume-center cohort of esophageal and esophagogastric junction cancer patients. We aimed to describe and analyze clinical key characteristics of patients with pCR compared with patients with non-complete pathological response (non-CR). The prognostic significance of pCR regarding survival probability, the impact of lymphnode metastases in patients with complete response of the primary tumor, the significance of signs of tumor regression in lymphnodes in patients with pCR, and the significance of postoperative completion of perioperative chemotherapy regarding survival probability were also evaluated.

## METHODS

### Patient selection

**Fig. 2:**
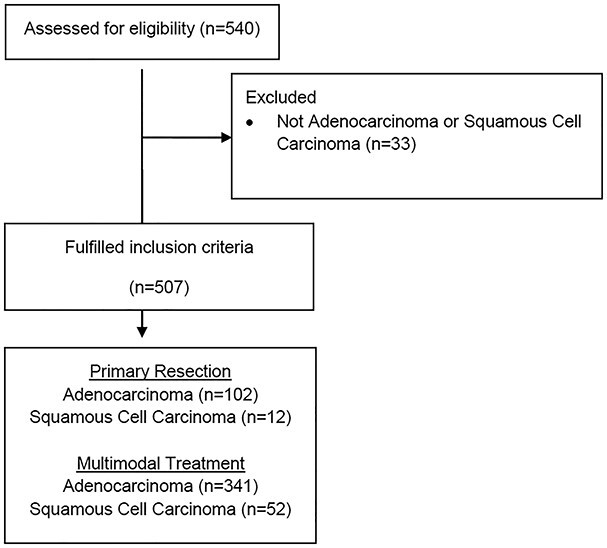
Study flow chart.

This study is reported with accordance to the STROBE-statement.^16^ Patient data from 01/2014 to 01/2021 were collected in our database for oncological upper gastrointestinal surgery (DRKS 00024369) and evaluated retrospectively. According to the study protocol, all consecutive patients with EC (ESCC and EAC) and adenocarcinoma of the esophagogastric junction (AEG)^17^ and adenocarcinoma with gastric location undergoing neoadjuvant treatment in curative intend and radical oncologic resection were included in this study. Out of 540 patients with upper gastrointestinal cancer, 393 patients with EC (341 with EAC, 52 with ESCC) were treated with neoadjuvant multimodal treatment in curative intend and therefore met the inclusion criteria for this study. A control cohort consisted of 102 patients with EAC and 12 patients with ESCC undergoing primary resection without neoadjuvant treatment (Fig. 2). The study was reviewed and approved by the Ethics Committee of the University of Freiburg (21-1093 and 21-1713).

The following data were extracted from our database: Demographic data: Age, Gender, Body-Mass-Index (BMI), RCS-Charlson-Comorbidity-Index;^18^ Disease-specific data: Histological Subtype of EC, Pretherapeutic TNM-Stage, Data concerning neoadjuvant and adjuvant treatment (neo−/adjuvant chemotherapy (nCTx)/nCTX-protocol; neo−/adjuvant chemoradiation (nCRT)/nCRT-protocol (nCT: Standard treatment FLOT-protocol according to;^19^ nCRT: Standard treatment CROSS-protocol according to ref. 7) Radiation dosage; time interval end of neoadjuvant treatment to surgery), assessment of tumor response after neoadjuvant treatment according to RECIST 1.1-guideline (Complete Response (CR), Partial Response (PR), Stable Disease (SD), Progressive Disease (PD));^20^ Surgical data: surgical procedure and perioperative data including overall complications according to the comprehensive complication index (CCI);^21^ Histopathological data: Postneoadjuvant TNM-Stage including residual tumor-classification (R-Status),^22^ Grading (G) of histopathological regression in the primary tumor bed according to Becker et al.;^23^ for the statistical analyses in this study, only patients with complete pathological response of the tumor in the primary tumor bed and in the harvested lymphnodes were classified as pathological complete responders (pCR), otherwise patients with histopathological regression grade 1a but ypT0ypN+ situation were classified as non-complete responders (non-CR) like other patients with incomplete response to neoadjuvant treatment. Furthermore, tumor-free lymphnodes in pCR-patients were assessed for signs of postneoadjuvant regression. In line with previous studies, (hyaline) fibrosis, acellular mucin and the presence of sheets of foamy histiocytes were considered as characteristic signs of tumor regression in the routine histopathological examination. Lymphnodes in patients with pCR were classified as negative lymphnodes without regressive signs (LN-/Reg-) and negative lymphnodes with regressive signs (LN-/Reg+)/Grade A according to Tsekrekos et al.^24^^,^^25^ (Supplementary Fig. S1). Survival data: Overall (OS) and disease-free survival (DFS).

### Statistical analysis

Survival data were systematically obtained from the cancer registry of our Comprehensive Cancer Center (CCCF). Follow-Up-data were collected until February 2022. Actuarial survival was calculated by univariable analysis using the Kaplan–Meier-Method, with log rank testing for comparison of subgroups. The median follow-up was 47 months calculated with Reverse Kaplan–Meier-Method.^26^ Results are expressed as Median (Interquartile Range) or as number (Percent). We used Mann–Whitney-U-test for descriptive analysis of non-parametric variables and Pearson’s chi-squared-test for categorical variables. Multivariate analyses were performed either by binary logistic regression or by Cox-Proportional-Hazard-Regression as applicable. Multiple Imputations were used to estimate missing data for multivariate analyses. Statistical analysis was performed using SPSS Version 28.0.1.0 (IBM Corp., Armonk, NY, USA) and R version 4.0.0 (R Foundation for Statistical Computing, Vienna, Austria) with R-Studio (R Studio Inc., Boston, MA, USA) and additional packages ggplot2 and survminer. Differences were considered statistically significant for *P* < 0.05.

## RESULTS

Details comparing the two cohorts with non-CR- vs. pCR-patients are shown in Tables 1 and 2 and in Supplementary Table 1, and 507 patients were included in this study according to the inclusion criteria (Fig. 2). A control cohort consisted of 102 patients with EAC and 12 patients with ESCC and primary resection of EC (Supplementary Table 2). Off the patients after neoadjuvant treatment, 393 patients were resected following neoadjuvant treatment (341 EAC and 52 ESCC). In the EAC-group, 48 patients (14.1%) had pCR. In the ESCC-group, 23 patients (44%) had pCR in the postoperative histopathological examination.

**Table 1:** Comparison of EAC-patients with neoadjuvant treatment

**Variable**		**non-CR (*n* = 293)**	**pCR (*n* = 48)**	***P*-value**
**Age (years)** (*n* = 341)		63.0 (55.0–71.00)	63.0 (57.5–72.8)	0.626^*^
**Gender**	Female	64 (22%)	14 (29%)	0.263#
	Male	229 (78%)	34 (71%)	
**BMI (Kg/m** ^**2**^ **)** (*n* = 341)		25.5 (23.1–28.1)	26.3 (23.8–28.7)	0.134^*^
**RCS Charlson-Index**	0	0 (0%)	0 (0%)	0.398#
	1	168 (57%)	25 (52%)	
	2	77 (26%)	17 (35%)	
	≥3	46 (17%)	6 (13%)	
**uT-Stage**	T0/Tis	2 (1%)	1 (2%)	0.534#
	T1	6 (2%)	1 (2%)	
	T2	34 (12%)	8 (17%)	
	T3	195 (66%)	29 (60%)	
	T4	30 (10%)	3 (6%)	
	NA	26 (9%)	6 (13%)	
**uN-Stage**	uN-	61 (21%)	10 (21%)	0.949#
	uN+	203 (69%)	33 (69%)	
	NA	29 (10%)	5 (10%)	
**cM-Stage**	cM0	254 (87%)	43 (90%)	0.775#
	cMX/1	39 (13%)	5 (10%)	
**Grading**	GX	114 (38%)	20 (42%)	0.559#
	G1	8 (3%)	3 (6%)	
	G2	87 (30%)	12 (25%)	
	G3	84 (29%)	13 (27%)	
**Neoadjuvant Chemotherapy**		248 (85%)	38 (79%)	0.339#
**Thereof: % FLOT**		239 (96%)	37 (97%)	
**Neoadjuvant Chemoradiation**		45 (15%)	10 (21%)	
**Thereof: % CROSS**		38 (84%)	7 (70%)	
**Radiation Dosage (Gy)** (*n* = 45)		41.4 (41.4–41.4)	41.4 (41.4–41.4)	0.969^*^
**Premature discontinuation of neoadjuvant treatment**		16 (6%)	4 (8%)	0.628#
**Days from end of neoadjuvant treatment to surgery** (*n* = 288)		43 (35–57)	43 (36–55)	0.850^*^
**RECIST 1.1**	CR/PR	128 (63%)	29 (81%)	0.045#
**Procedure**	2-Field-Esophagectomy	177 (60%)	37 (77%)	0.405#
	3-Field-Esophagectomy	2 (1%)	0 (0%)	
	Transhiatal-extended gastrectomy	32 (11%)	2 (4%)	
	Gastrectomy	37 (13%)	7 (15%)	
	Subtotal Gastrectomy	30 (10%)	1 (2%)	
	Gastrectomy+HIPEC	13 (5%)	1 (2%)	
	Esophagogastrectomy	1 (0%)	0 (0%)	
**CCI** (*n* = 341)		20.9 (0–40.6)	22.6 (0–39.7)	0.373^*^
**Length of Hospital Stay (days)** (*n* = 341)		14.0 (11.0–18.0)	15.0 (13.0–21.0)	0.069^*^
**Pathological T-Stage**	ypT0	8 (3%)	48 (100%)	<0.001#
	ypT1	66 (22%)	0 (0%)	
	ypT2	57 (20%)	0 (0%)	
	ypT3	138 (47%)	0 (0%)	
	ypT4	24 (8%)	0 (0%)	
**Pathological N-Stage**	ypN0	147 (50%)	48 (100%)	<0.001#
	ypN1	61 (21%)	0 (0%)	
	ypN2	34 (12%)	0 (0%)	
	ypN3	51 (17%)	0 (0%)	
**Tumor regression in lymphnodes in pCR-patients**	(LN-/Reg+)/Grade A	NA	22 (46%)	
	(LN-/Reg-)	NA	26 (54%)	
**Postneoadjuvant M-Stage**	yM0	256 (87%)	48 (100%)	0.009#
	ypM1	37 (13%)	0 (0%)	
**R-Status**	R0	278 (95%)	48 (100%)	0.109#
	R1	15 (5%)	0 (0%)	
**Postoperative UICC-Stage**	UICC-Stage 0	0 (0%)	48 (100%)	<0.001#
	UICC-Stage I	59 (20%)	0 (0%)	
	UICC-Stage II	92 (31%)	0 (0%)	
	UICC-Stage III	71 (24%)	0 (0%)	
	UICC-Stage IV	71 (24%)	0 (0%)	
**Histopathologic Regression**	Grade 1a - No residual tumor	8 (3%)	48 (100%)	<0.001#
	Grade 1b—Subtotal regression (<10% residual tumor)	92 (31%)	0 (0%)	
	Grade 2 – partial regression (10–50% residual tumor)	88 (30%)	0 (0%)	
	Grade 3 – no regression (>50% residual tumor)	95 (33%)	0 (0%)	
	Grade of regression not assessed	10 (3%)	0 (0%)	
**Adjuvant Treatment (% of patients receiving neoadjuvant CTx)**	Yes	161 (73%)	28 (74%)	0.882#

^*^Mann–Whitney-U-test

^#^Pearson’s chi squared-test

**Table 2:** Comparison of ESCC-patients with neoadjuvant treatment

**Variable**		**non-CR (*n* = 29)**	**pCR (*n* = 23)**	*P* **-value**
**Age (years)** (*n* = 52)		65 (55–72)	60 (56–68)	0.926^*^
**Gender**	Female	13 (45%)	5 (22%)	0.082#
	Male	16 (55%)	18 (78%)	
**BMI (Kg/m** ^**2**^ **)** (*n* = 52)		23.0 (21.1–27.0)	24.3 (22.5–26.6)	0.191^*^
**RCS Charlson-Index**	0	1 (3%)	1 (4%)	0.723#
	1	12 (41%)	13 (57%)	
	2	9 (31%)	5 (22%)	
	≥3	7 (24%)	4 (17%)	
**uT-Stage**	T1	2 (7%)	2 (9%)	0.767#
	T2	2 (7%)	4 (17%)	
	T3	18 (62%)	11 (48%)	
	T4	3 (10%)	3 (13%)	
	NA	4 (14%)	3 (13%)	
**uN-Stage**	uN-	3 (10%)	4 (17%)	0.675#
	uN+	22 (76%)	15 (65%)	
	NA	4 (14%)	4 (17%)	
**cM-Stage**	cM0	23 (79%)	21 (91%)	0.234#
	cMX/1	6 (21%)	2 (9%)	
**Grading**	GX	11 (38%)	12 (52%)	0.362#
	G1	0 (0%)	1 (4%)	
	G2	10 (34%)	7 (30%)	
	G3	8 (28%)	3 (13%)	
**Neoadjuvant Chemotherapy**		2 (7%)	0 (0%)	0.199#
**Thereof: % FLOT**		1 (50%)	0 (0%)	
**Neoadjuvant Chemoradiation**		27 (93%)	23 (100%)	
**Thereof: % CROSS**		23 (85%)	19 (83%)	
**Radiation Dosage (Gy)** (*n* = 47)		41.1 (41.1–41.4)	41.1 (41.1–41.4)	0.558^*^
**Premature discontinuation of neoadjuvant treatment**		2 (7%)	0 (0%)	0.199#
**Days from end of neoadjuvant treatment to surgery** (*n* = 51)		60 (45.5–84)	46.5 (37.75–59.75)	0.069^*^
**RECIST 1.1**	CR/PR	21 (72%)	18 (78%)	0.629#
**Procedure**	2-Field-Esophagectomy	25 (86%)	23 (100%)	0.064#
	3-Field-Esophagectomy	4 (14%)	0 (0%)	
	Transhiatal-extended gastrectomy	0 (0%)	0 (0%)	
	Esophagogastrectomy	0 (0%)	0 (0%)	
**CCI** (*n* = 52)		40.6 (26.1–60.2)	22.6 (0–62.6)	0.149^*^
**Length of Hospital Stay (days)** (*n* = 52)		23 (16–27.5)	16 (13–24)	0.069^*^
**Pathological T-Stage**	ypT0	4 (14%)	23 (100%)	<0.001#
	ypT1	6 (21%)	0 (0%)	
	ypT2	10 (34%)	0 (0%)	
	ypT3	8 (28%)	0 (0%)	
	ypT4	1 (3%)	0 (0%)	
**Pathological N-Stage**	ypN0	15 (52%)	23 (100%)	<0.001#
	ypN1	11 (38%)	0 (0%)	
	ypN2	3 (10%)	0 (0%)	
**Tumor regression in lymphnodes in pCR-patients**	(LN-/Reg+)/Grade A	NA	7 (30%)	NA
	(LN-/Reg-)	NA	16 (70%)	
**Postneoadjuvant M-Stage**	yM0	27 (93%)	23 (100%)	0.199#
	ypM1	2 (7%)	0 (0%)	
**R-Status**	R0	25 (86%)	23 (100%)	0.064#
	R1	4 (14%)	0 (0%)	
**Postoperative UICC-Stage**	UICC-Stage 0	0 (0%)	23 (100%)	<0.001#
	UICC-Stage I	7 (24%)	0 (0%)	
	UICC-Stage II	11 (38%)	0 (0%)	
	UICC-Stage III	8 (28%)	0 (0%)	
	UICC-Stage IV	3 (10%)	0 (0%)	
**Histopathologic Regression**	Grade 1a - No residual tumor	4 (14%)	23 (100%)	<0.001#
	Grade 1b – Subtotal regression (<10% residual tumor)	12 (41%)	0 (0%)	
	Grade 2 – partial regression (10–50% residual tumor)	6 (21%)	0 (0%)	
	Grade 3 – no regression (>50% residual tumor)	6 (21%)	0 (0%)	
	Grade of regression not assessed	1 (3%)	0 (0%)	

^*^Mann–Whitney-U-test

^#^Pearson’s chi squared-test

There were no significant differences regarding pCR-rate in EAC-patients between nCT and nCRT nor the interval between end of neoadjuvant treatment and surgery for both entities. The histological subtype (EAC vs. ESCC) was the only patient related clinical variable associated with an increased probability for pCR in univariate and multivariate analysis (OR 4.7, Supplementary Table 3). Postneoadjuvant CR- and PR-status according to RECIST1.1-guideline was associated with an OR of 1.9 in the prediction of pCR.

### Survival analysis

OS of the entire cohort was 61 months. Median OS was not reached in patients with primary surgery, and after neoadjuvant treatment, Median OS was 54 months (95%-CI: 40.1–67.9) with a non-significant difference between patients with EAC with 54 months (95%-CI: 38.5–69.5) and ESCC-patients with 35 months (95%-CI: 4.1–65.9 months) (*P* = 0.287).

OS was better in patients with pCR compared with non-CR-patients (Median OS—pCR: Median OS not reached vs. non-CR: 41 months (95%-CI: 30.3–51.7); *P* < 0.001). DFS also was better in pCR-patients (Median DFS—pCR: Median DFS not reached vs. 15.0 months (95%-CI: 12.5–17.5); *P* < 0.001). Similar observations were made for the two distinct histopathological entities separately as well (Supplementary Tables 4–7).

**Fig. 3:**
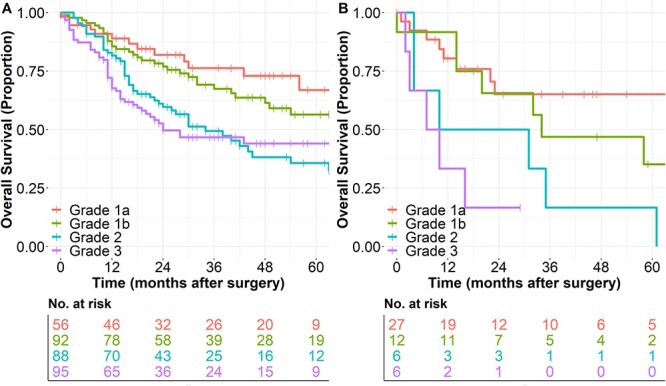
Overall survival with respect to the postneoadjuvant grade of histopathologic regression according to Becker et al. [23]. (A) in EAC patients after neoadjuvant treatment and (B) in ESCC-patients after neoadjuvant treatment.

**Fig. 4:**
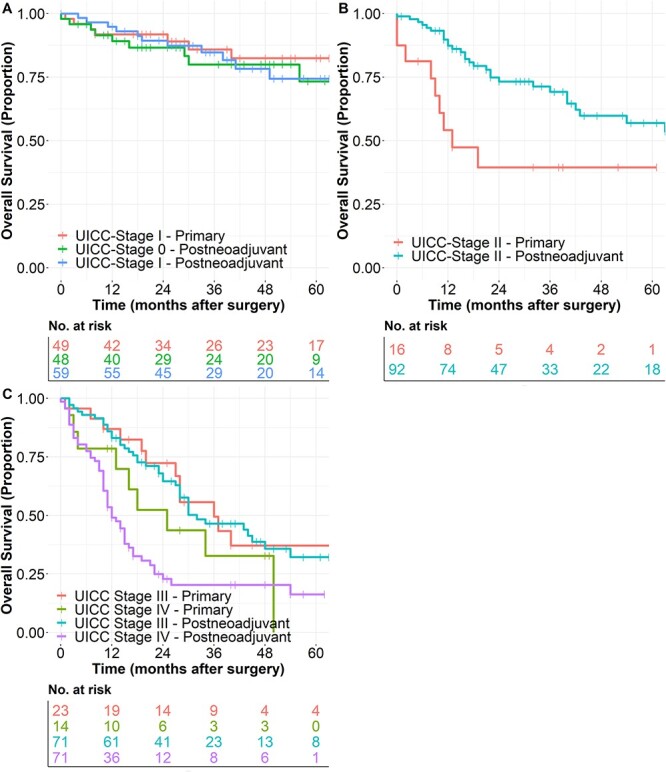
Comparison of overall survival-probability in EAC-patients according to postoperative UICC-Stage in patients after primary resection of EAC and following resection after neoadjuvant treatment of EAC. (A) UICC-Stage 0-I. (B) UICC-Stage II. (C) UICC-Stage III and IV.

In both entities, OS-probability was significantly associated with postneoadjuvant T-Stage. Another significant prognostic factor in univariate survival analysis was the postneoadjuvant N-Stage. Postneoadjuvant M-Stage and R-Status were as well associated with OS probability in univariate analysis. Postneoadjuvant histopathological grade of regression of the primary tumor was also associated with OS-probability. Similar observations were made regarding DFS for all analyses (Supplementary Tables 4–7; Fig. 3 and Supplementary Fig. S2). Multivariate analysis of prognostic factors with Cox-Regression-analysis revealed a difference in relevance of histopathological regression depending on the timing of survival prognosis. In the postneoadjuvant setting, only variables known from routine pretherapeutic staging and histopathological regression were used. In this scenario, the histological subtype, the pretherapeutic cM-Stage and the grade of histopathological regression were independent prognostic factors (Supplementary Table 8). Taking all postoperative and histopathological examinations into account, the relevance of the grade of histopathological regression vanished and the achieved postneoadjuvant tumor-stage (ypTNM-stage)—which to some extend contains the grade of histopathological regression—becomes the relevant prognostic factor besides tumor entity and surgical R-status (Table 3). This observation was confirmed, when survival data of postneoadjuvant patients with EAC were compared with the patients with EAC and primary resection without prior neoadjuvant treatment. For UICC stages I, III and IV, the survival of postneoadjuvant patients was similar to those with primary resection. In patients with UICC stage II, survival was better in postneoadjuvant patients. This observation demonstrates the necessity of neoadjuvant treatment in UICC-stage II-patients with EAC (Table 4, Fig 4A–C). The same analysis for ESCC-patients was not feasible due to the low number of patients in the primary resection-cohort.

**Table 3:** Cox-Regression-Analysis Postoperative Setting

**Variable**		**Hazard-Ratio**	**95%-CI**	*P* **-value**
**Histological Subtype**	EAC	Reference	Reference	0.084	
	ESCC	1.688	0.931–3.061		
**Pathological T-Stage**	ypT0-T2	Reference	Reference	0.013	
	ypT3-T4	1.837	1.134–2.976		
**Pathological N-Stage**	ypN0	Reference	Reference	0.002	
	ypN1-N3	2.046	1.293–3.240		
**Postneoadjuvant M-Stage**	ycM0	Reference	Reference	<0.001	
	ypM1	2.709	1.593–4.607		
**Pathological R-Status**	R0	Reference	Reference	<0.001	
	R1	4.195	2.141–8.220		

Excluded Variables by backwards stepwise variable selection: RCS-Charlson-Score, Pretherapeutic Grading and Grade of Histopathologic Regression.

**Table 4:** Comparison of overall survival-probability according to UICC-stage in patients after neoadjuvant treatment for EAC and after primary resection of EAC

**Postoperative UICC-Stage**	**Median OS**	**95%-CI**	***P*-value**
**UICC Stage 0**	Median OS not reached		0.489^*^ 0.853^*^^*^
**UICC Stage I – Primary**	Median OS not reached		0.317
**UICC Stage I – Postneoadjuvant**	Median OS not reached		
**UICC Stage II – Primary**	13.0	2.4–23.6	0.005
**UICC Stage II – Postneoadjuvant**	71.0	Not estimable	
**UICC Stage III – Primary**	36.0	19.5–52.5	0.860
**UICC Stage III – Postneoadjuvant**	32.0	18.2–45.8	
**UICC Stage IV – Primary**	25.0	10.5–39.5	0.222
**UICC Stage IV – Postneoadjuvant**	12.0	9.1–14.9	

^*^ vs. UICC-Stage I – Primary; ^*^^*^ vs. UICC-Stage I—Postneoadjuvant

### Prognostic significance of ypT0ypN+ stage

**Fig. 5:**
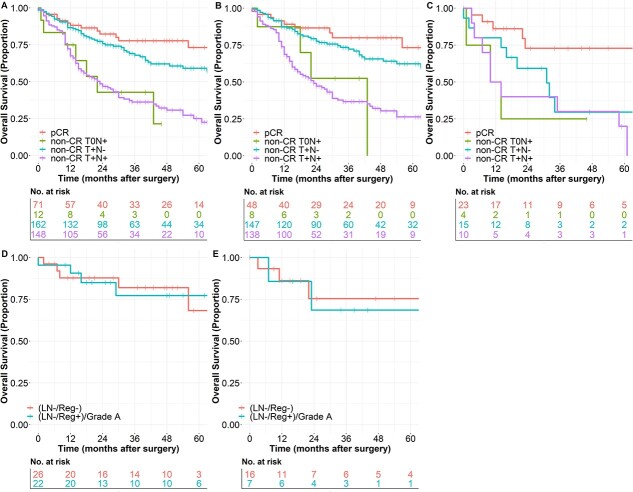
Significance of lymphatic metastases in patients with complete response of the primary tumor. Comparison of the different groups of patients. The best OS can be achieved in patients with pCR (ypT0ypN0). Residual tumor in lymphnodes in patients with complete response of the primary tumor (non-CR T0N+) is associated with a poor survival probability, which is comparable unfavorable as in patients with non-CR in the primary tumor-site and with lymphatic spread (non-CR T+N+). Patients without lymphnode metastases (non-CR T+N0) have only a slightly impaired OS-probability compared with patients with pCR. (A) All patients with EC after neoadjuvant treatment (EAC and ESCC combined) (Median OS—pCR: Median OS not reached, non-CR T0N+: 22.0 months (95%-CI: 10.4–33.5), non-CR T+N-: Median OS not reached, non-CR T+N+: 23.0 months (95%-CI: 15.3–30.7), *P*-values: pCR vs. non-CR T0N+: 0.001, pCR vs. non-CR T+N-: 0.055, pCR vs. non-CR T+N+: <0.001, non-CR T0N+ vs. non-CR T+N-: 0.016; non-CR T0N+ vs. non-CR T+N+: 0.956; non-CR T+N- vs. non-CR T+N+: <0.001), (B) in patients with EAC (Median OS—pCR: Median OS not reached, non-CR T0N+: 43.0 months (95%-CI: not estimable), non-CR T+N-: Median OS not reached, non-CR T+N+: 23.0 months (95%-CI: 15.8–30.2), *P*-values: pCR vs. non-CR T0N+: 0.025, pCR vs. non-CR T+N-: 0.129, pCR vs. non-CR T+N+: <0.001, non-CR T0N+ vs. non-CR T+N-: 0.108; non-CR T0N+ vs. non-CR T+N+: 0.868; non-CR T+N- vs. non-CR T+N+: <0.001) and (C) in patients with ESCC (Median OS—pCR: Median OS not reached, non-CR T0N+: 10.0 months (95%-CI: 0.0–22.7), non-CR T+N-: 31.0 months (95%-CI: 14.3–47.7), non-CR T+N+: 10.0 months (95%-CI: 2.8–17.2), *P*-values: pCR vs. non-CR T0N+: 0.017, pCR vs. non-CR T+N-: 0.042, pCR vs. non-CR T+N+: <0.002, non-CR T0N+ vs. non-CR T+N-: 0.419; non-CR T0N+ vs. non-CR T+N+: 0.827; non-CR T+N- vs. non-CR T+N+: <0.380). pCR (ypT0ypN0) patients with signs of postneoadjuvant regression in lymphnodes have the same OS-probability compared with patients without signs of regression in the examined lymphnodes (*P* = 0.975). (D) In patients with EAC (*P* = 0.919). (E) In patients with ESCC (*P* = 0.828).

A key aim of this study was to evaluate the prognostic effect of local T-stage and lymphatic postneoadjuvant N-stage separately. While patients with pCR show the best oncologic outcome, patients with ypT0-situation but lymphatic metastases (non-CR T0N+) have a devastating prognosis. Interestingly, patients without complete response of the primary tumor, but without lymphatic metastasis (non-CR T+N−) have almost the same favorable prognosis as pCR-patients, while patients with local non-CR and lymphatic metastases (non-CR T+N+) have a significantly worse prognosis. This observation was made for EAC-patients and ESCC-patients separately as well, even though the effect was less distinct in ESCC-patients (Fig. 5A–C). This observation lays the emphasis on the prognostic significance of the postneoadjuvant N-stage. Non-CR T0N+ is associated with a comparably unfavorable prognosis as non-CR T+N+. The best overall survival probability is achieved in patients with pCR followed by non-CR patients with negative postneoadjuvant lymphnode-status.

The presence of lymphnodes with signs of postneoadjuvant regression (LN-/Reg+)/Grade A in patients with pCR did not significantly influence overall (All neoadjuvant patients: Median OS—both groups: Median OS not reached; *P* = 0.975, EAC-patients: *P* = 0.919; ESCC-patients: *P* = 0.828; Fig. 5D and E) and disease-free survival probability (All neoadjuvant patients: Median DFS—both groups: Median DFS not reached; *P* = 0.521; EAC-patients: *P* = 0.332; ESCC-patients: *P* = 0.708) compared with patients with negative lymphnodes (LN-/Reg-).

### Necessity of postneoadjuvant completion of chemotherapy

Differing from neoadjuvant chemoradiation according to the CROSS-protocol, in which the multimodal treatment ends with the surgical treatment and patients are reffered to oncologic follow-up examinations thereafter, neoadjuvant chemotherapy according to the FLOT-protocol includes neoadjuvant/preoperative and adjuvant/postoperative chemotherapy. Under various cicumstances, some patients do not receive the planned postoperative chemotherapy cycles due to patients will, perioperative complications or other reasons. To evaluate the necessity of postneoadjuvant completion of chemotherapy in patients with pCR-status, we evaluated 38 patients with EAC undergoing neoadjuvant chemotherapy. Off these patients, 28 received postoperative/adjuvant chemotherapy and the other 10 patients did not. While both groups had good survival probability, a trend toward a better overall survival probability could be observed in patients with postoperative completion of chemotherapy (*P* = 0.062; Supplementary Fig. S3). To interpret this observation, a non-significant difference in CCI-Scores has to be considered (no adjuvant chemotherapy: CCI 25.7 (10.45–49.9), with adjuvant chemotherapy: CCI 22.6 (0.0–37.0); *P* = 0.519). Although different reasons for non-completion of chemotherapy might bias this observation, it at least gives a hint that postoperative completion of chemotherapy offers survival advatages even in patients with pCR.

## DISCUSSION

In this study, we described the clinical characteristics of patients with pCR compared with non-CR-patients with EC and AEG. Improved overall and disease-free survival were observed in patients with pCR compared with non-CR in the entire cohort, and for both distinct histological entities individually, in univariate analysis. Multivariate analysis revealed that the histopathological grade of regression was not an independent predictor for prolonged survival, instead the achieved postneoadjuvant ypTNM-stage was the important predictor for improved overall survival together with the histological subtype of EC and the postoperative resection status. Remaining lymphatic metastases in patients with complete response of the primary tumor imply a devastating prognostic impact. Only if true complete response with ypT0 and also with ypN0 is reached, patients have a favorable prognosis. Otherwise, patients with ypT0 but lymphatic metastasis have the same unfavorable prognosis as patients with other T-stages and lymphatic spread of disease. According to our data, postoperative resumption of perioperative chemotherapy might further improve survival probability even in patients with pCR.

The observed postneoadjuvant pCR-rates are within the expectable range with 15% for EAC and 44% for ESCC.^5^^,^^7^ Off note, the length of the interval between the end of neoadjuvant treatment and the surgical resection of the tumor did not influence the probability for pCR in our cohort, in neither EAC nor ESCC-patients. This topic is currently discussed and incongruent observations are made by several authors.^27–29^ Prediction of pCR was not possible by means of the routine clinical data. Only a correlation between postneoadjuvant response evaluation according to RECIST 1.1-criteria and pCR could be observed in our cohort. A reliable prediction of pCR was not possible with routine restaging examinations, as it had to be expected, based on the results of previous studies. A meta-analysis found that accuracy of standard-endoscopic biopsies, endoscopic ultrasound and PET-CT as single modalities was insufficient for detection of residual disease after nCRT for EC.^30^ The recently published preSANO-trial demonstrated that the diagnostic accuracy can be improved significantly by combination of endoscopy with deep-(bite-on-bite) biopsies, endoscopic ultrasound with fine-needle aspiration cytology of suspicious lymphnodes and PET-CT. While 31% of patients were classified false-negatively as clinical complete responders by standard re-staging examinations, the diagnostic accuracy was increased with the above mentioned modalities of the preSANO-protocol to only 11% false-negative patients. In addition, PET-CT detected distant interval metastases in 10% of patients after nCRT, which prevented unnecessary esophagectomy.^31^

Our study demonstrated that OS and DFS were improved in patients with major response (Grade 1a and 1b) compared with patients with minor response (Grade 2 and 3) to neoadjuvant treatment. pCR-patients also had better survival probability than non-CR-patients in univariate analysis, in the entire cohort and for both distinct entities in particular. This observation is in line with previous studies.^11^^,^^29^ Moreover, previous studies found that survival of patients with non-response to neoadjuvant treatment was not different from primary esophagectomy, while neoadjuvant treatment was associated with specific treatment-related toxicity.^32^^,^^33^ These observations both imply a need for better pretherapeutic prediction models of tumor response prior to neoadjuvant treatment to avoid unnecessary treatment-associated toxicity and surgical delay on the one hand and to guide therapy for patients with anticipated good clinical response to treatment on the other hand.

In addition, the individual factors of the TNM-system including the R-status were confirmed to be of prognostic significance in our study. Multivariate Cox-Regression-analyses were performed in two settings in our study. Without the knowledge of the ypTNM stages, the grade of regression is an individual predictor for survival probability. However, in the second multivariate analysis including the postoperative and postneoadjuvant ypTNM-stage, the effect of the grade of regression vanishes and only the individual factors of the achieved postneoadjuvant tumor-stage is important for prediction of survival probability besides R-status and the histopathological subtype of the tumor. This means that the degree of pathological ‘downstaging’ is more important than the grade of regression for prognostic evaluation. This observation is confirmed in our data, as grade 1a- and grade 1b-regression do not have significantly different survival probabilities.

Regarding the significance of lymphatic spread in patients with complete response of the primary tumor (non-CR T0N+), we were able to demonstrate that this pathological finding was associated with a poor prognosis, which did not differ from any other patient with lymphnode metastases. This observation was also made by other authors recently in a post-hoc analysis of a Chinese prospective trial including ESCC-patients,^34^ as well as in a dutch population-based registry study in EC-patients.^29^ Furthermore, our study demonstrated that patients with supposed pretherapeutic lymphatic spread could achieve similar survival probability as patients without lymphatic metastases, if complete remission can be achieved in lymphnodes as well (Fig. 5D and E). Whether a histopathological examination that exceeds routine assessment of regressive changes in lymphnodes after neoadjuvant treatment can improve prognosis of survival probability in patients with pCR requires further elucidation in future studies.^35^

The observation that patients with non-CR T0N+ have the same unfavorable prognosis as patients with non-CR T+N+ reinforces the important prognostic effect of the postneoadjuvant N-stage. Moreover, the observation clarifies the necessity of explicit identification of non-CR T0N+ patients in organ-preserving-treatment concepts in complete clinical responders with surveillance and surgery only as needed. These concepts are evaluated in the prospective randomized SANO (NTR6803), ESOSTRATE (NCT02551458) and ESORES (preliminary registration identifier: DRKS 00022801)-trial.^36^

Our data suggest that postoperative continuation of perioperative chemotherapy offers improved survival. As mentioned before, this observation is supposedly biased by a higher degree of perioperative complications in patients without postoperative chemotherapy, which itself impairs the oncologic prognosis.^37–39^ Further studies—ideally prospectively randomized controlled trials—are needed to investigate the need for postoperative chemotherapy in patients with pCR.

Limitations of the current study include of course the retrospective design of the study, although we tried to minimize bias within this study. The problem of selection bias was addressed by definition of distinct selection criteria for inclusion of patients from the prospectively maintained database with consecutive patients. As demonstrated in Tables 1 and 2, completeness of data was high. In rare cases of missing data, multiple imputations were used to address this issue. As this study is of retrospective nature, no specific clinical response evaluation (similar to the preSANO-protocol) other than routine re-staging examinations were available. The preoperative diagnostic accuracy of pCR-detection therefore cannot be assessed within this study. Although retrospective data have its limitations, especially in times of novel diagnostic and treatment modalities—as ‘surveillance and surgery as needed’ or novel therapeutic options as immunotherapy for EC—the value of retrospective data is high. New hypotheses for future randomized trials can be generated due to retrospective observation of patients in new treatment concepts. With immunotherapy and other advances of perioperative multimodal treatment of EC, raising rates of pCR after neoadjuvant treatment can be expected in the future. The importance of this observation and possibly of future diversification of treatment options due to pCR will therefore increase.^40^

In conclusion, the grade of postneoadjuvant response and particularly the achieved postneoadjuvant ypTNM-stage influences the survival probability of patients with EC and AEG-cancer. Patients with complete response of the primary tumor site, but lymphnode metatases with vital tumor cells (non-CR T0N+), have a dismal prognosis, which corresponds to the prognosis of stage III-patients. Only true pathological complete response with ypT0ypN0-stage offers superior survival probabilities. The possibilities of organ-preserving concept with surveillance and surgery as needed in complete responders will hopefully offer further improvement for patients with EC in the future.

## Supplementary Material

Supplementary_DataR1_doac095
