# Supplementary material for: Pathological complete response in multimodal treatment of esophageal cancer: a retrospective cohort study
Source: Dis Esophagus. 2022 Dec 27;36(7):doac095. doi: 10.1093/dote/doac095 (PMC10317005; doi:10.1093/dote/doac095)
Supplement: Supplementary_DataR1_doac095 [file supplementary_datar1_doac095.docx]

**Pathological complete response in multimodal treatment of esophageal cancer – a cohort study**

Julian Hipp, Jasmina Kuvendjiska, Hans Christian Hillebrecht, Sylvia Timme-Bronsert, Stefan Fichtner-Feigl, Jens Hoeppner and Markus K. Diener

**Figure S1:** Representative histopathological images of

1. an esophagus and the primary tumor of an EAC without signs of postneoadjuvant regression (Grade 3).
2. an esophagus with postneoadjuvant pathological complete response of the primary tumor (Grade 1a).
3. a lymphnode metastasis with sign of regression and with signs of vital tumor (Grade B).
4. a lymphnode with postneoadjuvant fibrosis as a sign of postneoadjuvant regression, but without vital tumor cells (LN-/Reg+)/Grade A).

**
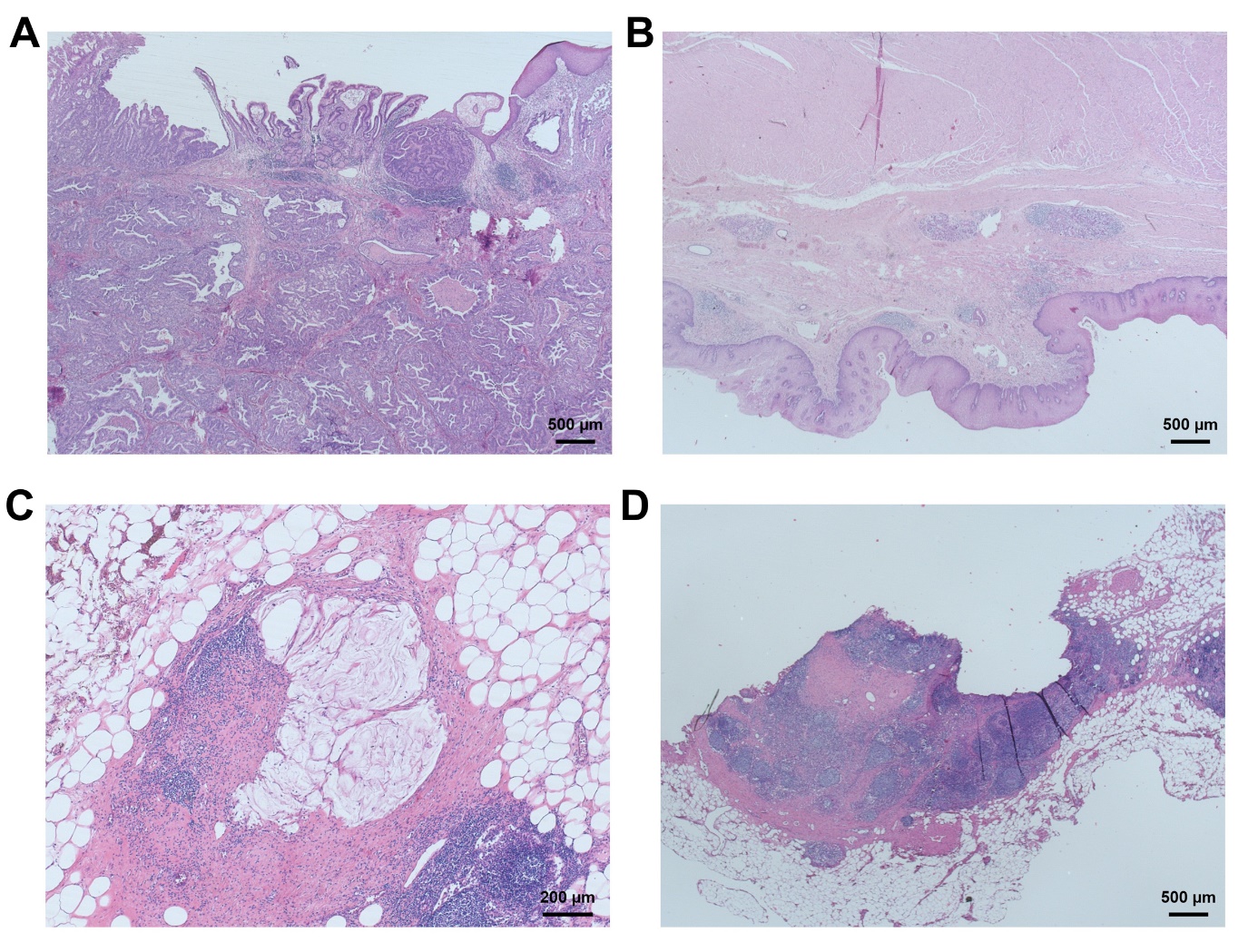
**

**Figure S2:** Overall survival analysis of the entire cohort with respect to

| **A)** ypT-stage in EAC-patients | **B)** ypT-stage in ESCC-patients |
| --- | --- |
| **C)** ypN-stage in EAC-patients | **D)** ypN-stage in ESCC -patients |
| **E)** Postneoadjuvant M-stage in EAC-patients | **F)** Postneoadjuvant M-stage in ESCC -patients |
| **G)** Residual tumor-classification (R-status) in EAC-patients | **H)** Residual tumor-classification (R-status) in ESCC -patients |


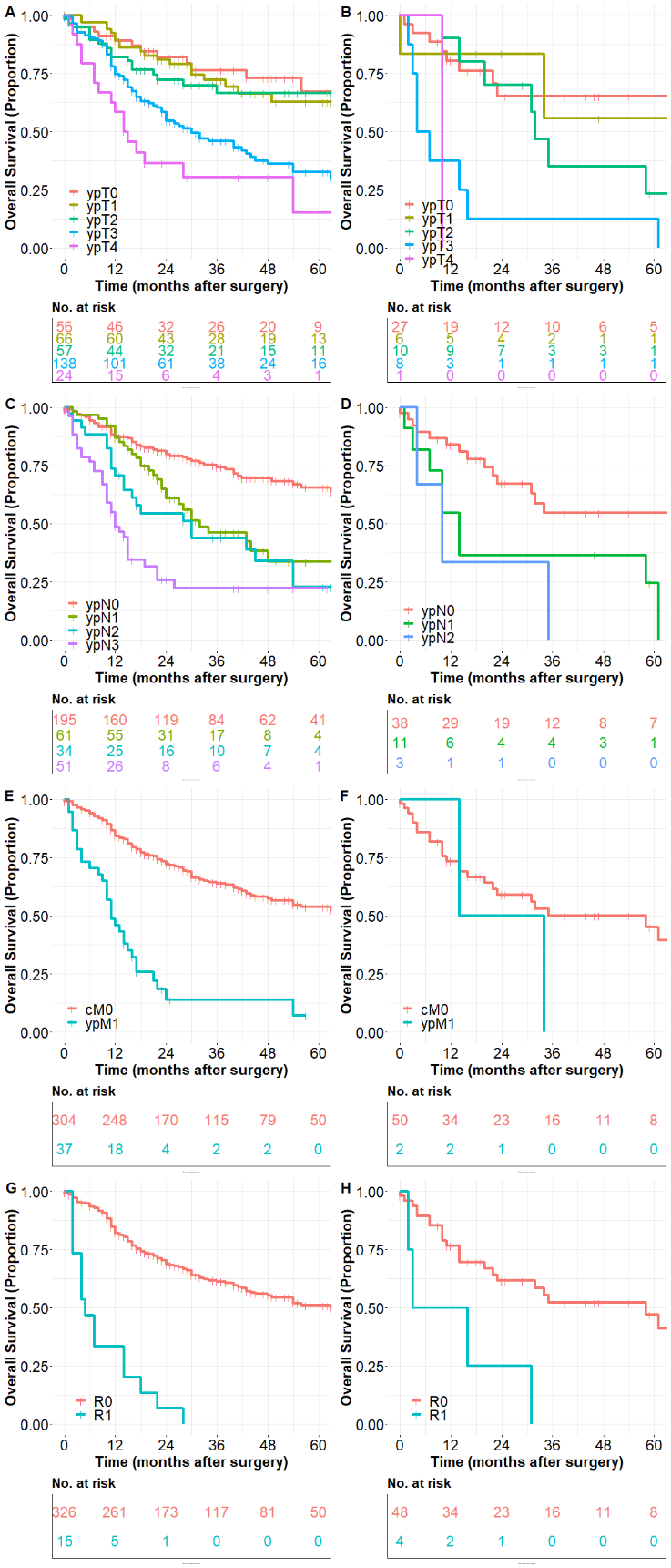


**Figure S3:** Comparison of patients the necessity of postneoadjuvant continuation of perioperative chemotherapy accoring to the FLOT-protocol in patients with pCR-status. While both groups had good survival probability with not estimable median OS, a treand towards a better overall survival probability could be observed in patients with postoperative completion of chemotherapy (p=0.062).


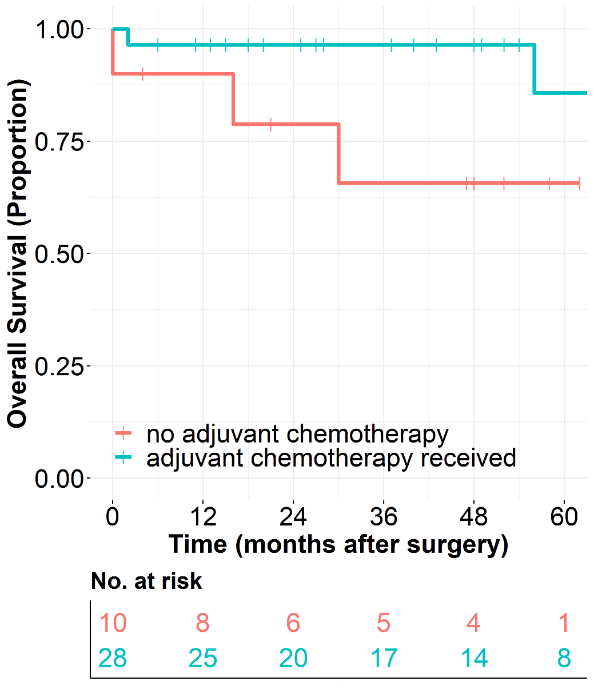


**Supplementary data Table 1: Entire cohort of patients with neoadjuvant treatment**

| **Variable** | | **non-CR (n=322)** | **pCR (n=71)** | **p-value** |
| --- | --- | --- | --- | --- |
| **Age (years)** | | 63.0 (55.0-71.0) | 62.0 (57.0-72.0) | 0.757* |
| **Gender** | Female | 77 (24%) | 19 (27%) | 0.613# |
|  | Male | 245 (76%) | 52 (73%) |  |
| **BMI (Kg/m²)** | | 25.3 (22.8-28.1) | 25.9 (23.6-28.4) | 0.354* |
| **RCS Charlson-Index** | 0 | 1 (0%) | 1 (1%) | 0.544# |
|  | 1 | 180 (56%) | 38 (54%) |  |
|  | 2 | 86 (27%) | 22 (31%) |  |
|  | ≥3 | 55 (17%) | 10 (14%) |  |
| **Histological subtype** | EAC | 293 (91%) | 48 (68%) | <0.001# |
|  | ESCC | 29 (9%) | 23 (32%) |  |
| **uT-Stage** | T0-2 | 46 (16%) | 16 (26%) | 0.059# |
|  | T3-4 | 246 (84%) | 46 (74%) |  |
| **uN-Stage** | uN- | 64 (22%) | 14 (23%) | 0.940# |
|  | uN+ | 225 (78%) | 48 (77%) |  |
| **cM-Stage** | cM0 | 277 (86%) | 64 (90%) | 0.563# |
|  | cMX/1 | 45 (14%) | 7 (10%) |  |
| **Grading** | G1-2 | 105 (53%) | 23 (59%) | 0.516# |
|  | G3 | 92 (47%) | 16 (41%) |  |
| **Neoadjuvant Chemotherapy** | | 250 (78%) | 38 (54%) | <0.001# |
| **Neoadjuvant Chemoradiation** | | 72 (22%) | 33 (46%) |  |
| **Premature discontinuation of neoadjuvant treatment** | | 18 (6%) | 4 (6%) | 0.988# |
| **Days from end of neoadjuvant treatment to surgery** | | 44.0 (36.0-59.0) | 44.0 (37.0-56.5) | 0.974* |
| **RECIST 1.1** | CR/PR | 195 (61%) | 53 (77%) | 0.015# |

* Mann-Whitney-U-test

# Pearson’s chi squared-test

**Supplementary data Table 2: Control cohort consisting of patients with EAC and ESCC with primary resection of EC.**

| **Variable** | | **EAC-primary**  **(n=102)** | **ESCC-primary**  **(n=12)** |
| --- | --- | --- | --- |
| **Age (years)** | | 73 (63-80.3) | 69.5 (64.5-78.25) |
| **Gender** | Female | 32 (31%) | 6 (50%) |
|  | Male | 70 (69%) | 6 (50%) |
| **BMI (Kg/m²)** | | 26.3 (23.6-29.5) | 23.9 (19.7-26.2) |
| **RCS Charlson-Index** | 0 | 0 (0%) | 1 (8%) |
|  | 1 | 41 (40%) | 3 (25%) |
|  | 2 | 22 (22%) | 4 (33%) |
|  | ≥3 | 39 (38%) | 4 (33%) |
| **Grading** | G1-2 | 39 (59%) | 5 (71%) |
|  | G3 | 27 (41%) | 2 (29%) |
| **Pathological T-Stage** | pT0 | 4 (4%) | 0 (0%) |
|  | pT1 | 47 (46%) | 7 (58%) |
|  | pT2 | 18 (18%) | 3 (25%) |
|  | pT3 | 22 (23%) | 2 (17%) |
|  | pT4 | 11 (11%) | 0 (0%) |
| **Pathological N-Stage** | pN0 | 61 (60%) | 8 (67%) |
|  | pN1 | 19 (19%) | 2 (17%) |
|  | pN2 | 13 (13%) | 2 (17%) |
|  | pN3 | 9 (9%) | 0 (0%) |
| **M-Stage** | cM0 | 98 (96%) | 12 (100%) |
|  | pM1 | 4 (4%) | 0 (0%) |
| **R-Status** | R0 | 94 (92%) | 12 (100%) |
|  | R1 | 8 (8%) | 0 (0%) |
| **UICC-Stage** | UICC-Stage I | 49 (48%) | 6 (50%) |
|  | UICC-Stage II | 16 (16%) | 3 (25%) |
|  | UICC-Stage III | 23 (23%) | 3 (25%) |
|  | UICC-Stage IV | 14 (14%) | 0 (0%) |
| **Adjuvant Treatment** | Yes | 23 (23%) | 0 (0%) |

**Supplementary data table 3:** Multivariate Analysis of preoperative factors predicting pathological complete response.

| **Variable** | | **Odds-Ratio** | **95%-CI** | **p-value** |
| --- | --- | --- | --- | --- |
| **Histological Subtype** | EAC | Reference | Reference | <0.001 |
|  | ESCC | 4.724 | 2.503-8.916 |  |
| **RECIST 1.1** | SD/PD | Reference | Reference | 0.042 |
|  | CR/PR | 1.906 | 1.025-3.545 |  |

Excluded Variable by backwards stepwise variable selection: Type of neoadjuvant treatment (nCT or nCRT)

**Supplementary data table 4:** Univariate analysis of overall survival according to postneoadjuvant ypTNM-stage and histopathologic grade of regression in EAC-patients.

| **Variable** | | **Median OS (months)** | **95%-CI** | **p-value compared to reference** |
| --- | --- | --- | --- | --- |
| **Pathological T-Stage** | ypT0 | Median OS not reached | - | Reference |
|  | ypT1 | Median OS not reached | - | 0.440 |
|  | ypT2 | Median OS not reached | - | 0.445 |
|  | ypT3 | 30.0 | 19.6-40.4 | <0.001 |
|  | ypT4 | 14.0 | 8.3-19.7 | <0.001 |
| **Pathological N-Stage** | ypN0 | Median OS not reached | - | Reference |
|  | ypN1 | 32.0 | 16.7-47.3 | <0.001 |
|  | ypN2 | 30.0 | 13.8-46.2 | <0.001 |
|  | ypN3 | 12.0 | 8.2-15.8 | <0.001 |
| **Postneoadjuvant M-Stage** | ycM0 | 67.0 | - | <0.001 |
|  | ypM1 | 11.0 | 7.4-14.6 |  |
| **R-Status** | R0 | 63.0 | - | <0.001 |
|  | R1 | 5.0 | 1.8-8.2 |  |
| **Histopathologic Regression** | Grade 1a - No residual tumor | Median OS not reached | - | Reference |
|  | Grade 1b-3 | 44.0 | 31.9-56.1 | 0.001 |
|  | Grade 1b – Subtotal regression (<10% residual tumor) | 67.0 | - | 0.202 |
|  | Grade 2 – partial regression (10–50% residual tumor) | 34.0 | 21.1-46.9 | 0.001 |
|  | Grade 3 – no regression (>50% residual tumor) | 24.0 | 3.0-44.9 | <0.001 |

**Supplementary data table 5:** Univariate analysis of overall survival according to postneoadjuvant ypTNM-stage and histopathologic grade of regression in ESCC-patients

| **Variable** | | **Median OS (months)** | **95%-CI** | **p-value compared to reference** |
| --- | --- | --- | --- | --- |
| **Pathological T-Stage** | ypT0 | Median OS not reached | - | Reference |
|  | ypT1 | Median OS not reached | - | 0.967 |
|  | ypT2 | 32.0 | 26.4-37.6 | 0.226 |
|  | ypT3 | 4.0 | 0.3-7.7 | <0.001 |
|  | ypT4 | 10.0 | - | 0.068 |
| **Pathological N-Stage** | ypN0 | Median OS not reached | - | Reference |
|  | ypN1 | 14.0 | 8.5-19.5 | 0.018 |
|  | ypN2 | 10.0 | 0.4-19.6 | 0.041 |
| **Postneoadjuvant M-Stage** | ycM0 | 35.0 | 0-72.7 | 0.336 |
|  | ypM1 | 14.0 | - |  |
| **R-Status** | R0 | 58.0 | 25.6-90.4 | 0.005 |
|  | R1 | 3.0 | 0-16.7 |  |
| **Histopathologic Regression** | Grade 1a - No residual tumor | Median OS not reached | - | Reference |
|  | Grade 1b-3 | 20.0 | 0-47.0 | 0.003 |
|  | Grade 1b – Subtotal regression (<10% residual tumor) | 34.0 | - | 0.345 |
|  | Grade 2 – partial regression (10–50% residual tumor) | 10.0 | - | 0.010 |
|  | Grade 3 – no regression (>50% residual tumor) | 7.0 | - | 0.005 |

**Supplementary data table 6:** Univariate analysis of disease-free survival according to postneoadjuvant ypTNM-stage and histopathologic grade of regression in EAC-patients

| **Variable** | | **Median DFS (months)** | **95%-CI** | **p-value compared to reference** |
| --- | --- | --- | --- | --- |
| **Pathological T-Stage** | ypT0 | 56.0 | 17.8-94.1 | Reference |
|  | ypT1 | 37.0 | 27.8-46.2 | 0.473 |
|  | ypT2 | 18.0 | 12.6-23.4 | 0.049 |
|  | ypT3 | 13.0 | 10.7-15.3 | <0.001 |
|  | ypT4 | 8.0 | 2.1-13.9 | <0.001 |
| **Pathological N-Stage** | ypN0 | 40.0 | 31.5-48.5 | Reference |
|  | ypN1 | 14.0 | 9.6-18.4 | <0.001 |
|  | ypN2 | 12.0 | 8.6-15.4 | <0.001 |
|  | ypN3 | 9.0 | 5.9-12.1 | <0.001 |
| **Postneoadjuvant M-Stage** | ycM0 | 22.0 | 14.3-29.7 | <0.001 |
|  | ypM1 | 8.0 | 4.6-11.4 |  |
| **Histopathologic Regression** | Grade 1a - No residual tumor | 56.0 | 17.8-94.2 | Reference |
|  | Grade 1b-3 | 15.0 | 12.3-17.7 | <0.001 |
|  | Grade 1b – Subtotal regression (<10% residual tumor) | 32.0 | 13.3-50.7 | 0.128 |
|  | Grade 2 – partial regression (10–50% residual tumor) | 15.0 | 12.5-17.5 | <0.001 |
|  | Grade 3 – no regression (>50% residual tumor) | 12.0 | 9.1-14.9 | <0.001 |

**Supplementary data table 7:** Univariate analysis of disease-free survival according to postneoadjuvant ypTNM-stage and histopathologic grade of regression in ESCC-patients

| **Variable** | | **Median DFS (months)** | **95%-CI** | **p-value compared to reference** |
| --- | --- | --- | --- | --- |
| **Pathological T-Stage** | ypT0 | Median DFS not reached | - | Reference |
|  | ypT1 | 34.0 | 0-84.9 | 0.900 |
|  | ypT2 | 16.0 | 10.2-21.8 | 0.135 |
|  | ypT3 | 4.0 | 0.2-7.8 | 0.003 |
|  | ypT4 | 2.0 | - | 0.011 |
| **Pathological N-Stage** | ypN0 | 22.0 | 4.5-39.5 | Reference |
|  | ypN1 | 10.0 | 4.8-15.2 | 0.093 |
|  | ypN2 | 4.0 | 0.8-7.2 | 0.021 |
| **Postneoadjuvant M-Stage** | ycM0 | 16.0 | 5.9-26.1 | 0.012 |
|  | ypM1 | 14.0 | - |  |
| **Histopathologic Regression** | Grade 1a - No residual tumor | Median DFS not reached | - | Reference |
|  | Grade 1b-3 | 10.0 | 5.2-14.8 | 0.007 |
|  | Grade 1b – Subtotal regression (<10% residual tumor) | 17.0 | - | 0.304 |
|  | Grade 2 – partial regression (10–50% residual tumor) | 4.0 | - | 0.011 |
|  | Grade 3 – no regression (>50% residual tumor) | 7.0 | - | 0.015 |

**Supplementary data table 8:** Cox-Regression-Analysis - Postneoadjuvant Setting

| **Variable** | | **Hazard-Ratio** | **95%-CI** | **p-value** | |  |
| --- | --- | --- | --- | --- | --- | --- |
| **Histological Subtype** | EAC | Reference | Reference | | 0.065 | |
|  | ESCC | 1.800 | 0.964-3.364 | |  |  |
| **Pretherapeutic cM-Stage** | cM0 | Reference | Reference | | 0.006 | |
|  | cMX/1 | 2.093 | 1.235-3.549 | |  |  |
| **Histopathologic Regression** | Grade 1b-3 | Reference | Reference | | 0.003 | |
|  | Grade 1a | 0.295 | 0.133-0.654 | |  |  |

Excluded Variables by backwards stepwise variable selection: RCS-Charlson-Score, Pretherapeutic Grading, Pretherapeutic uT- and uN-Stage
